# Influence of *Mikania micrantha* Kunth Flavonoids on Composition of Soil Microbial Community

**DOI:** 10.3390/ijms26010064

**Published:** 2024-12-25

**Authors:** Qilin Yang, Wenyang Cui, Zijun Guan, Zhenzhen Wang, Israt Jahan, Ping Li, Feng Qin, Xi Qiao, Bo Liu, Jian Yan

**Affiliations:** 1 Key Laboratory of Agro-Environment in Tropics, Ministry of Agriculture and Rural Affairs, Guangdong Engineering Research Centre for Modern Eco-Agriculture and Circular Agriculture, College of Natural Resources and Environment, South China Agricultural University, Guangzhou 510642, China; 2 Shenzhen Branch, Guangdong Laboratory of Lingnan Modern Agriculture, Genome Analysis Laboratory of the Ministry of Agriculture and Rural Affairs, Agricultural Genomics Institute at Shenzhen, Chinese Academy of Agricultural Sciences, Shenzhen 518120, Chinaqiaoxi@caas.cn (X.Q.);

**Keywords:** *Mikania micrantha*, flavonoids, soil microbial communities, arbuscular mycorrhizal fungi (AMF), invasive plant management

## Abstract

*Mikania micrantha*, one of the world’s most destructive invasive species, is known for causing significant ecological and economic harm. While extensive research has focused on its growth characteristics, secondary metabolites, and control measures, its chemical interactions with the environment—particularly the role of flavonoids in shaping soil microbial communities—remain underexplored. In this study, we identified and quantified ten flavonoids from *M. micrantha* root exudates using UPLC-MS, including Hispidulin, Isorhamnetin, and Mikanin. To examine their impact, crude flavonoid extracts were applied to soil in potted experiments, which demonstrated that these compounds significantly increased soil fungal diversity and boosted the relative abundance of arbuscular mycorrhizal fungi (AMF). Furthermore, KEGG pathway analysis revealed that flavonoid addition elevated the copy numbers of genes involved in nitrogen cycling and metabolic functions, enhancing nutrient availability and microbial activity. Additionally, crude flavonoid extracts promoted the relative abundance of beneficial soil bacteria, such as Achromobacter, as well as AMF, both of which contribute to nutrient acquisition, plant growth, and soil health. These findings indicate that *M. micrantha*’s flavonoids can alter soil microbial community composition, thereby creating a favorable environment that reinforces its competitive edge over native plants.

## 1. Introduction

*Mikania micrantha* is a vine plant belonging to the Asteraceae family’s *Mikania* genus [1]. As one of the 100 worst invasive weeds in the world, it induces serious ecological damage [2,3] and economic loss [4]. With high genetic diversity [4], fast growth, and strong reproduction, *M. micrantha* invade successfully due to its natural advantages [5]. It can inhibit the photosynthesis of the host [6,7], and form nutritional competition with native species. Understanding secondary metabolic components will help us explore its invasion mechanism easily. Numerous studies have demonstrated that *M. micrantha* contains a wide variety of beneficial secondary metabolites such as lactones [8], flavonoids [9], organic acids, phenols [10], and steroids, leading to changes in the physical and chemical properties of the soil [11], enzyme activities, functional gene expression [4], and soil microbial biomass [12].

Flavonoids are a class of polyphenolic compounds in plants, which have antioxidant, anti-cancer, anti-AIDS, antibacterial, anti-allergic, anti-inflammatory and other effects [13]. Flavonoids’ secondary metabolites are found abundantly in plants, particularly in the form of glycosides and aglycones [14]. They are involved in various biological processes, including plant development, stress response, and defense mechanisms against pathogens [15]. In the soil, flavonoids are primarily introduced via root exudates, leachates, and the decomposition of plant litter, where they undergo complex transformations due to microbial activity [16]. Soil microbes, especially bacteria and fungi, can metabolize these compounds, influencing their bioavailability and functional properties [16].

Flavonoids are typically metabolized by soil microorganisms through processes like glycosylation, hydroxylation, methylation, and reduction [17]. These microbial transformations can alter the structure and activity of flavonoids, affecting their interactions with other soil organisms [16]. Previous research has shown that flavonoids can act as signaling molecules in plant–microbe interactions, with certain soil bacteria capable of utilizing these compounds as a carbon source [10]. In addition, flavonoids have been reported to influence the microbial community by selectively promoting the growth of beneficial microorganisms, such as nitrogen-fixing bacteria and mycorrhizal fungi, while inhibiting the growth of pathogens [18]. This selective microbial response plays a crucial role in shaping the structure and function of soil microbial communities [19].

The soil environment is a premise for plant growth, and is responsible for providing adequate nutrition, suitable physical and chemical properties, and a soil microbial community [20,21]. When flavonoids are introduced into the soil, they can alter these factors, influencing the structure and composition of microbial communities [19]. Flavonoids can modify microbial enzyme activities, including those involved in the degradation of organic matter and nutrient cycling, which in turn influences soil fertility and plant growth [22]. The ability of flavonoids to modulate soil microbial communities and enzyme activities has been widely recognized as an important mechanism through which plants, including invasive species like *M. micrantha*, alter soil ecosystems [12]. These effects can lead to shifts in microbial diversity, potentially enhancing the plant’s ability to thrive in competitive or stressed environments [23].

The flavonoid compounds typically enter the soil through the natural processes of plant litter decomposition and root exudation. However, in many cases, it remains challenging to directly link specific active compounds to their particular effects on different soil organisms. Limited research has been conducted on the mechanisms through which flavonoids from *M. micrantha* influence soil. This study aims to address this gap by investigating the role of flavonoids produced by *M. micrantha* in modulating soil microbial communities. To achieve this, we utilized advanced chromatographic techniques (UPLC-MS) to identify and quantify flavonoids from *M. micrantha* root exudates. Potted soil experiments were designed to evaluate how these flavonoids influence microbial diversity, community composition, and functional potential. By exploring these interactions, this research seeks to uncover the chemical mechanisms underlying *M. micrantha*’s influence on soil ecology, contributing to a deeper understanding of invasive species’ ecological strategies and their potential environmental impacts.

## 2. Results

### 2.1. M. micrantha Aerial Part Flavonoids

**Figure 1:**
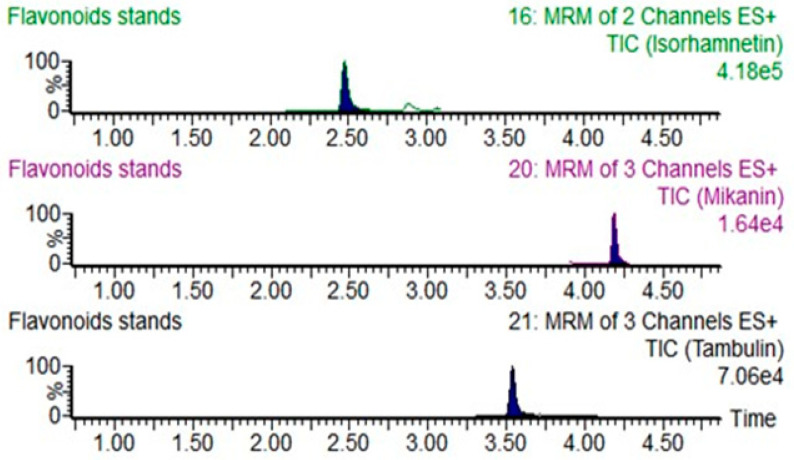
The chromatogram of flavonoids quantified by UPLC-MS, which involved the co-injection of the sample and the standard for analysis.

**Figure 2:**
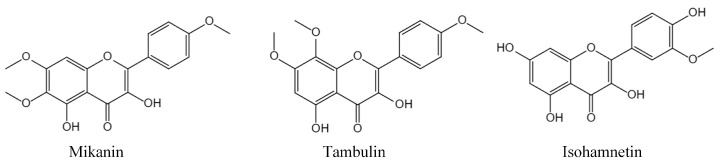
Structure identification of flavonoids by their ^1^H-NMR. Mikanin; Tambulin; Isohamnetin.

To characterize the flavonoid compounds in *M. micrantha*, the chemical structures of compounds 1–3 were elucidated using NMR and HR-ESI-MS techniques (Figure 1), and we compared the NMR data to the references. These compounds were identified as Mikanin (1), Tambulin (2), and Isohamnetin (3). Their purity was confirmed to exceed 95%, as analyzed by UPLC-MS (Figure 2), along with ^1^H-NMR and ^13^C-NMR spectroscopy. The detailed spectroscopic data for these known flavonoids samples are as follows (Supplementary Figures S1–S5).

Mikanin (1): Yellow powder, ESI-MS *m*/*z* 345.32 [M + H]^−^, ^1^H NMR (600 MHz, DMSO) δ 12.39 (1H, s, H-5), 9.62 (1H, s, H-2), 8.19 (1H, d, J = 9.0 Hz, H-3′4′), 7.13 (1H, d, J = 9.0 Hz, H-2′5′), 6.92 (1H, s, H-8). 3.93 (3H, s, Me-11), 3.85 (3H, s, Me-10), 3.74 (3H, s, Me-12) [24].

Tambulin (2): Yellow powder, ^1^H NMR was similar with Mikanin, ESI-MS was same as Mikanin, the two were isomers of each other. ^1^H-NMR (600 MHz, DMSO) δ 12.62 (1H, s, H-5), 8.25 (1H, d, *J* = 8.8 Hz, H-3′4′), 7.06 (1H, d, *J* = 9.0 Hz, H-6), 6.90 (1H, d, *J* = 8.8 Hz, H-2′5′). 3.92 (3H, s, Me-12), 3.86 (3H, s, Me-11), 3.73 (3H, s, Me-10). ^13^C NMR (151 MHz, DMSO) δ 162.06 (C-7), 133.15 (C-2), 131.07 (C-8), 114.16 (C-3′4′), 63.50 (C-11), 60.48 (s, C-10), 55.87 (C-12).

Isohamnetin (3): Yellow powder, C_16_H_12_O_7_, Ω = 11, ^1^H-NMR (600 MHz, DMSO) δ 13.09 (1H, s, H-5), 7.41 (1H, dd, J = 8.3, 2.3 Hz, H-5′), 7.39 (1H, d, J = 2.2 Hz, H-2′), 6.88 (1H, d, J = 8.3 Hz, H-6′), 6.66 (1H, s, H-8), 6.55 (1H, s, H-6), 3.74 (3H, m, Me-10) [25].

### 2.2. M. micrantha Root Exudates Flavonoids

**Figure 3:**
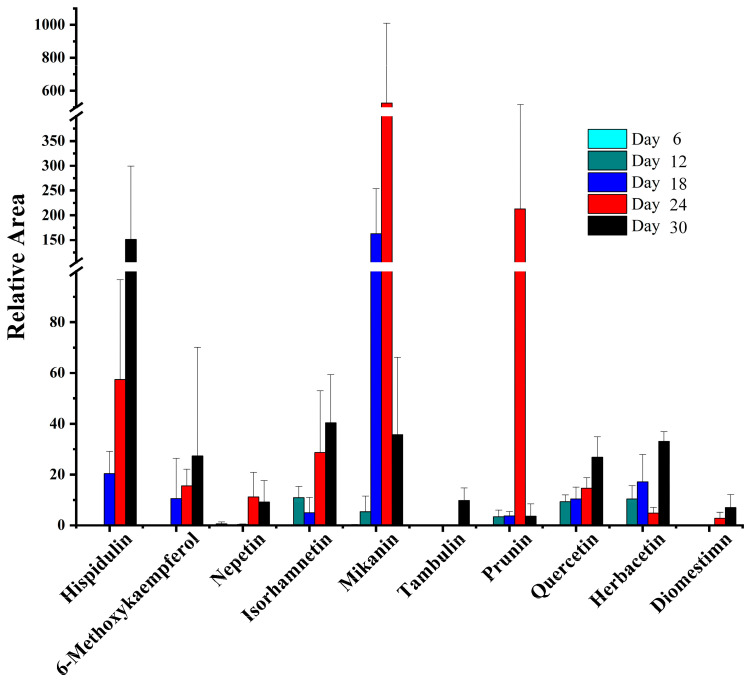
UPLC-MS analysis of *M. Micrantha* root exudates. Compare the differences of flavonoids in culture for 6 days, 12 days, 18 days, 24 days, and 30 days. Biological repeat n = 5.

The contents of flavonoids increased over cultivation time. Isorhamnetin, Mikanin, Prunin, Quercetin, and Herbacetin could be detected after 6 days, and the others could only be detected after 12 days. The content change of Prunin over 30 days was significant (Figure 3). By 24 days, the content had increased, prominently, but it suddenly decreased at 30 days. We hypothesize that prunin, a 7-O glycoside of naringenin, may reach a threshold concentration, at which point root-associated microorganisms metabolize the glycoside component for nutrients, leaving naringenin, a key precursor for various flavonoids and isoflavonoids, as the remaining structure. In the data tested, prunin content appeared a significant area (17,391.95) in a single test at day 24, which may be a threshold point.

### 2.3. Impact of Crude Flavonoids on Soil Microbial Populations and Metabolism

**Figure 4:**
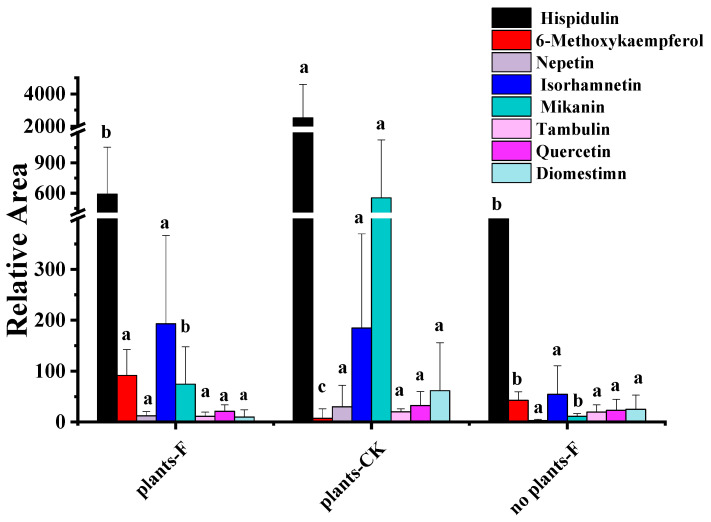
Content changes of flavonoids in the pot experiment. Plants-CK: plants with no additives. Plant-F: plants with flavonoids. No plant-F: no plants with flavonoids. Different letters indicate significant differences between the two groups, and the same letters indicate no significant difference between the two groups.

The evaluation of crude flavonoids in cultivable soil bacteria showed no significant differences in bacterial growth (OD_600_) or flavonoid concentration compared to the control group. Therefore, flavonoid treatment did not alter the 16S rRNA profiles. Compared to plant-F, the flavonoid content was higher in plants-CK, suggesting that the added compounds were metabolized and utilized by soil microorganisms. By the end of the experiment, most of the added flavonoids had been catabolized and consumed (Figure 4). These results indicate that flavonoids create favorable conditions for the accumulation of soil microorganisms capable of metabolizing such compounds, enabling a rapid microbial response to maintain soil homeostasis.

### 2.4. Changes of Abundance and Diversity of Soil Microorganisms

**Figure 5:**
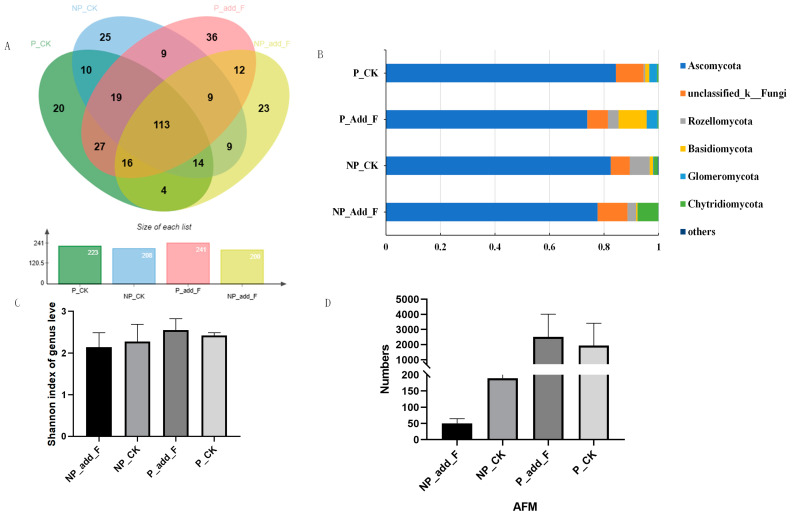
ITS sequencing results. (**A**) The soil fungi community of each treatment group at the level of the genus. (**B**) The soil fungi community of each treatment group at the level of the phylum. Others represent species with abundance less than 1%. (**C**) α-diversity (Shannon index) of soil fungi community; P_Add_F—plants with added flavonoids; NP_Add_F—no plants with flavonoids; NP_CK—no plants with no additives; P_CK—plants with no additives; (**D**). FUNGuild function prediction analysis, comparison of the relative abundances of Arbuscular mycorrhizal fungi in different treatment groups.

To evaluate changes in soil microbial abundance and diversity, 16S rRNA and ITS sequencing were used to track fungal and bacterial communities across treatment groups. The analysis of fungal diversity revealed that 113 fungal genera were shared among the four treatments (Figure 5A). The P_Add_F group contained 36 unique fungal species, followed by the NP_CK group with 25 unique species, and the NP_Add_F and P_CK groups with 23 and 20 unique species, respectively. This suggests that adding flavonoids enhances fungal community diversity in the soil. A fungal genus-level classification graph is shown in the Supplementary Material (Figure S6A).

Based on the Shannon index, there was no significant difference in fungal alpha diversity between bare soil and soil planted with *Mikania micrantha*, indicating that *M. micrantha* invasion alone did not alter soil fungal diversity (Figure 5C). However, in *M. micrantha*-planted soil, flavonoid addition significantly increased fungal diversity. Compared to the P_CK group, the crude flavonoid extract treatment raised the relative abundances of *Rozellomycota* and *Basidiomycota* (Figure 5B).

Arbuscular mycorrhizal fungi (AMF), which aid in nutrient cycling and nitrogen and phosphorus uptake, had a higher relative abundance in *M. micrantha*-invaded soil than in non-invaded soil (Figure 5D). This increase may be linked to the nutrient demands generated by rapid *M. micrantha* growth. In the invaded soil, AMF abundance was slightly higher in the flavonoid-treated group than in the control, suggesting that flavonoid compounds also support AMF growth. The effect of *M. micrantha* invasion on AMF abundance is similar to that of flavonoid addition, implying that flavonoids may contribute to the success of *M. micrantha* invasion.

### 2.5. Effects of M. micrantha and Flavonoids on Soil Bacterial Diversity and Nitrogen Cycling

**Figure 6:**
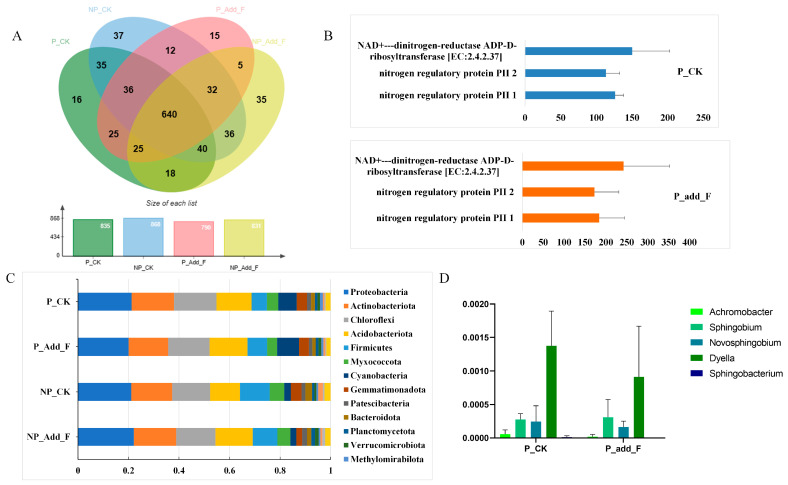
16S rRNA sequencing of soil bacteria. (**A**). The bacterial community composition of each treatment group at the genus level. (**B**) Comparison of the gene copy numbers of homologous proteins related to nitrogen metabolism. (**C**) Bacterial composition at the phylum level in each treatment group. (**D**) Comparison of the relative abundances of 5 bacterial genera in different treatment groups of invasive soil. *Achromobacter*, *Sphingobacterium*, *kroppenstedtia*, *Novosphingobium*, *Sphingobium*.

At the genus level, 640 bacterial species were shared across treatment groups (Figure 6A). The NP__CK and NP_Add_F groups contained 37 and 35 unique species, respectively, while the P_CK and P_Add_F groups included 16 and 15 unique species. *Sphingobacterium* was present only in the P_CK and NP_F treatment groups, while *Sphingobacterium*, *Kroppenstedtia*, *Novosphingobium*, and *Sphingobium* were not detected in LB medium experiments, likely due to the complex soil environment, where microbial interactions can lead to mutual promotion or inhibition. A bacterial genus-level classification graph is shown in the Supplementary Materials (Figure S6B).

In LB medium experiments, flavonoid addition increased the relative abundance of *Kroppenstedtia* (Figure 6B). Analyzing bacterial composition at the phyla level (Figure 6C), flavonoid addition slightly increased the relative abundance of Cyanobacteria across all plant treatment groups. Additionally, *M. micrantha* invasion reduced the abundance of *Bacteroidota* and *Desulfobacterota*, with flavonoid addition further decreasing their levels. This suggests a synergistic influence of *M. micrantha* invasion and flavonoid addition on soil microbial communities, potentially mediated by specific compounds released during the invasion.

During the analysis of the KEGG metabolic pathway, it was found that the two types of homologous proteins were all related to nitrogen regulation (Figure 6D), suggesting that the invasion of *M. micrantha* can promote the nitrogen cycle in the soil. This result corresponds to the previous report [26] that *M. micrantha* invasion can promote the nitrogen cycle in the soil.

### 2.6. Functional Analysis of KEGG Pathways

**Figure 7:**
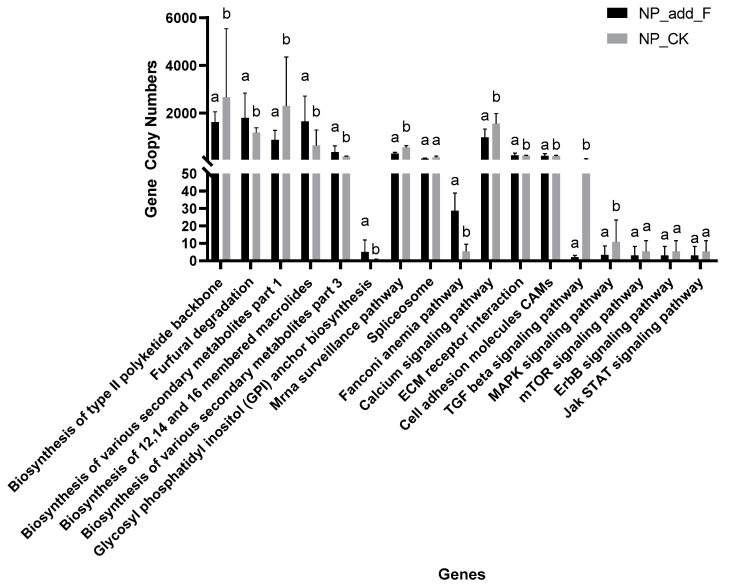
KEGG functional predictive analysis of Pathway Level 3. NP-F: Noninvasive soil with crude flavonoids extract. NP-CK: Noninvasive soil without addition. One-way ANOVA was used to assess differences between groups. Different letters indicate significant differences between the treatment groups (*p* ≤ 0.05), and the same letters indicate no significant difference between the groups, n = 5.

The KEGG pathway analysis (Figure 7) indicates that adding crude flavonoids to noninvasive soil can significantly influence the abundance of functional gene copies. Specifically, flavonoid extracts increased gene counts related to the biosynthesis of 12, 14, and 16 membered macrolides, the biosynthesis of various secondary metabolites (part 3), glycosyl phosphatidyl inositol (GPI) anchor biosynthesis, the Fanconi anemia pathway, ECM receptor interaction and cell adhesion molecules (CAMs). Conversely, flavonoid addition reduced gene counts involved in type II polyketide backbone biosynthesis, furfural degradation, the biosynthesis of various secondary metabolites (part 1), mRNA surveillance, calcium signaling, TGF-beta signaling, and MAPK signaling pathways.

## 3. Discussion

*Mikania micrantha*, a highly invasive species, significantly disrupts ecosystems by overpowering native plants [27]. However, its root exudates contain bioactive compounds, including newly identified flavonoids like Mikanin, which may offer therapeutic potential. These flavonoids are not only beneficial for the plant itself, enhancing its growth through interactions with soil microorganisms, but they may also shape the soil environment to support its invasion. This dual role emphasizes the need to understand both its ecological impacts and potential benefits, particularly through its flavonoid-rich root exudates (Figure 1 and Figure 2).

Our findings reveal that the invasion and flavonoid contributions of *M. micrantha* to soil microbial communities correlate with increased soil flavonoid content, including compounds such as Isorhamnetin, Mikanin, Prunin, Quercetin, and Herbacetin (Figure 3). Notably, Prunin showed a unique fluctuation in concentration, suggesting that root-associated microorganisms may metabolize it, leaving naringenin as a precursor for other flavonoids. Other flavonid compounds like isorhamnetin, mikanin quercetin, and herbacetin produce glycosides that bolster antioxidant defenses, offering protection against UV radiation, drought, and herbivory [28,29,30]. Overall, these results align with previous research highlighting flavonoid glycosides’ roles in shaping soil microbial interactions and stability [31].

Flavonoids in plant root exudates play a key role in regulating plant growth and shaping soil microbial communities. For example, in legumes, flavonoids facilitate symbiotic relationships with nitrogen-fixing rhizobia [32]. Similarly, our study found that crude flavonoid extracts from *M. micrantha* significantly altered the composition of soil microbial communities, as revealed by 16S rRNA and ITS sequencing (Figure 5). This aligns with Hassan and Mathesius [33], who showed that flavonoids act as root–rhizosphere signaling molecules, influencing plant–microbe interactions. Previous studies also suggest flavonoids serve as allelochemicals, affecting soil microbial dynamics and altering fungal and bacterial compositions [34,35,36].

The diversity of fungal communities was notably higher in flavonoid-treated soil, with an increased abundance of *Rozellomycota* and *Basidiomycota* (Figure 5). These groups, including arbuscular mycorrhizal fungi (AMF), are vital for nutrient cycling, particularly nitrogen and phosphorus uptake, supporting the notion that flavonoids in *M. micrantha* root exudates promote AMF growth. Tian [37], who found that flavonoids like quercetin enhance AMF colonization, aiding the plant’s invasion by improving nutrient cycling. Additionally, Chen Liang [38] suggested that endemic bacteria in invaded areas of *M. micrantha* are linked to unculturable bacteria within the γ-Proteobacteria and α-Proteobacteria, indicating a complex microbial response influencing soil nutrient cycling and plant health.

Furthermore, flavonoids appear to influence broader soil processes, such as carbon, nitrogen, and sulfur cycling, and the decomposition of organic matter [16]. The presence of *Cyanobacteria*, *Basidiomycota*, and *Desulfobacterota* in flavonoid-treated soils suggests that flavonoids may also affect soil structure and microbial interactions beyond nutrient cycling (Figure 6). These microbial shifts may contribute to the successful invasion of *M. micrantha* by fostering conditions favorable to the plant’s growth. Yu Hanxia [27] found that *M. micrantha* invasion promotes the growth of nitrogen cycle-related microbes, such as ammonia-oxidizing archaea (AOA) and ammonia-oxidizing bacteria (AOB), enhancing soil nitrogen availability to support plant growth. Cyanobacteria [39,40] and Basidiomycota [41] play important roles in soil carbon cycling and soil structure formation. Desulfobacterota [42] is involved in sulfate reduction and is related to sulfur cycling. Together, these findings suggest that flavonoids play a critical role in these microbial transformations, highlighting their importance in plant–microbe interactions.

The predictive KEGG pathway analysis (Figure 7) further reveals the functional implications of flavonoid addition. We observed an increase in genes related to the biosynthesis of macrolides, secondary metabolites, and GPI anchor biosynthesis, all of which are crucial for bioactive compound production and microbial interactions. The upregulation of genes associated with ECM receptor interaction and cell adhesion molecules (CAMs) suggests that flavonoids may enhance microbial colonization and communication in the rhizosphere. In contrast, pathways related to stress response and signaling, including type II polyketide backbone biosynthesis, were downregulated, reflecting a shift in microbial metabolic priorities. This indicates that flavonoid exposure may reprogram microbial community functions, supporting the plant’s success by promoting nutrient cycling and ecosystem interactions. While this study highlights the influence of flavonoids on soil microbial communities and *M. micrantha* invasion, the underlying mechanisms remain unclear. Future research should explore the cellular and molecular processes by which flavonoids influence microbial abundance, diversity, and behavior. A deeper understanding of these interactions will provide valuable insights into the role of flavonoids in the ecological success of *M. micrantha*, and their impacts on soil health and microbial dynamics.

## 4. Materials and Methods

### 4.1. Materials

*M. micrantha* plant: collected in Jiulong Town, Huangpu District, Guangzhou City (23°22′15.79″ N. 113°28′6.26″ E), and identified as a *M. micrantha* plant belonging to the Asteraceae by Dr. Haijun Yang from South China Agricultural University.

Collection of soil samples from *M. mikania* invasion: the 5 *M. mikania* invasion sites were Zhongluotan Town, Huangpu District, Guangzhou City (ZLT) (23°24′4.43″ N. 113°25′36.26″ E), South China Agricultural University Wetland, Zhongluotan Town, Huangpu District, Guangzhou City Park (SCAU) (23°09′24.66″ N. 113°20′59.04″ E), Enping (EP) (22°04′34.30″ N. 112°13′52.60″ E), Jiulong Town, Huangpu District, Guangzhou City (JLZ) (23°22′15.79″ N. 113°28′6.26″ E), Shenzhen (SZ) (22°35′59.26″ N. 114°29′18.52″ E).

### 4.2. Extraction and Isolation of Flavonoids from M. micrantha

To extract flavonoids, 35 kg of powdered aerial parts of *M. micrantha* were naturally dried, extracted three times with 95% ethanol (Pubo Instrument Co., Ltd., Guangzhou, China), and concentrated under reduced pressure at 40 °C, yielding an ethanol crude extract. This extract was dissolved in deionized water and sequentially partitioned three times in a 1:1 volume with petroleum ether (General-reagent^®^, Shanghai, China), chloroform (General-reagent^®^, Shanghai), ethyl acetate (General-reagent^®^, Shanghai, China), and n-butanol (Enox^®^ TBPB, Suzhou, China), resulting in three extraction layers. The chloroform extract was then subjected to silica gel column chromatography and eluted with an acetone/petroleum ether gradient, yielding 40 fractions (P1–P40). Compounds 1 and 2 were identified by TLC. The ethyl acetate extract was mixed with silica gel (200 mesh) in a 1.5:1 ratio, loaded onto a silica gel column, and eluted with a petroleum ether/ethyl acetate gradient (10:0 to 0:10) to obtain 15 fractions (G1–G15). Selected fractions (G14 and G15) were further purified on an MCI column (Mitsubishi Chemical, Shanghai, China) using a methanol/water gradient. Fraction G14 was de-pigmented, washed with 70% methanol, and subjected to gel chromatography (methanol/dichloromethane = 1:1). High-purity compound 3 (17 mg) was isolated at a 254 nm wavelength by HPLC (Agilent Technologies Co., Ltd., Santa Clara, CA, USA).

### 4.3. Root Exudate Collection and Flavonoid Quantification

Due to the low flavonoid concentrations in invasive soils, root exudates were collected to examine flavonoid content. Aerial parts of *M. micrantha* were collected from the Wetland Park of South China Agricultural University. Stem branches (20 cm) with two leaves were cultured in deionized water under foil to encourage rooting at 28 °C and 70–80% humidity. Once rooted, branches were transferred to 1/2-strength MS medium diluted 10-fold, and replaced every 3 days over a 30-day period. Exudate samples were collected on days 6, 12, 18, 24, and 30, with five replicates for each period. Samples were lyophilized, dissolved in methanol, concentrated under vacuum, and re-dissolved in 1.5 mL chromatography-grade methanol. After filtering through a 0.22 um PETE filter (), samples were stored in brown vials.

### 4.4. Quantitative Analysis of Flavonoids by UPLC-MS

Quantitative analysis was conducted on an ACQUITY™ UPLC system coupled with a Xevo TQD mass spectrometer, using an ACQUITY UPLC^®^ BEH C18 column (2.1 mm × 50 mm, 1.7 um) (Waters Inc., Milford, MA, USA) maintained at 40 °C. The auto-sampler was conditioned at 25 °C with a 2 uL injection volume. Mass spectrometric detection was performed with electrospray ionization (ESI) in positive and negative modes, using capillary voltages of 3.0 KV and 2.22 KV, respectively, and a source temperature of 150 °C. The collision gas (Ar) and desolvation gas (N₂) were set at 400 °C with a flow rate of 800 L/h, and cone gas was set at 50 L/h. Multiple reaction monitoring (MRM)-optimized compounds with a dwell time of 0.025 s were used. TargetLynx v4.1 software was used to analyze the data. Gradient elution was performed with 0.1% formic acid in water (A) and acetonitrile (D) as follows: 0–1.0 min, 80% A; 1.0–4.0 min, 50–5% A; 4.0–6.0 min, 5% A; 6.0–6.5 min, 5–80% A; 6.5–8.0 min, 80% A. The flow rate was set to 0.3 mL/min.

### 4.5. Influence of Crude Flavonoids on Soil Microorganisms

Fresh aerial parts of *M. micrantha* were extracted with chloroform for 30 s, filtered with medium-speed filter paper to remove insoluble impurities, and concentrated under vacuum and reduced pressure at 40 °C to obtain a chloroform extract. After continuous leaching and enrichment, 7 g of chloromethane extract was obtained and separated by polyamide column chromatography to isolate flavonoids. In polyamide column chromatography, the elution starts with 3 L of pure water to remove highly polar compounds. This is followed by increasing ethanol concentrations: 30% (4 L), 50% (4 L), 70% (4 L) and 90% (3 L), and ends with 100% ethanol (4 L). Every 500 mL was collected as a fraction and then dried. After quantitative determination by UPLC-MS, the fractions with the main component flavonoids were combined into the crude extracts of flavonoids. The crude extracts were saved for subsequent activity experiments and addition experiments.

### 4.6. Influence of Crude Flavonoids on Cultivable Soil Bacteria

The invasive soil was taken from *M. micrantha* invasive areas from a depth of 1–3 cm in the surface layer, naturally dried, and passed through a 30-mesh sieve to remove the plant litter. A 1 g soil sample was dissolved in sterile water and cultured at 28 °C and 180 rpm for 30 min. After incubating for 15 min, it was transferred to a sterile centrifuge tube and centrifuged at 25 °C 10,000 rpm for 30 s. A 100 uL soil microbial extract, diluted 1000 times, was shaken and cultured at 28 °C 180 rpm in 30 mL LB medium with 50 ppm flavonoids, dissolved with DMSO. We recorded the OD_600_ and concentration changes of flavonoids in medium at 0 h, 12 h, 20 h, 36 h, 48 h and 60 h. The sample at 60 h was also observed for bacterial abundance and diversity by 16S rRNA.

### 4.7. Effects of Crude Flavonoids on Soil Microorganisms

The aerial parts of *M. micrantha* were collected from the Wetland Park of South China Agricultural University and cut into 20 cm branches at stem nodes. Each branch was preserved in 2 leaves for photosynthesis, cultured in deionized water, and covered with tin foil to promote rooting at 28 °C and 70–80% humidity. After 3–5 days, the branches took root and were transplanted into polyethylene flower pots with a diameter of 12 cm (1.0 kg of soil, 3 branches per pot). The cultivation soil was non-invasive soil collected in Jiulong Town, Guangzhou City. Except for the negative control, the other flowerpots were covered with plastic mesh to prevent the litter of the plants from entering the potting soil, but at the same time, this did not affect the air permeability of the soil. After 10 days, the plants were successfully planted in pots, and their growth was rendered stable. Five treatments were set up, as follows: (1) After planting *M. micrantha*, allow it to grow naturally, and separate the underground part from the above-ground part using a net to prevent litter from entering the soil. (2) Add leaves—Add 5.0 g of Mikania leaves, dried at 60 °C and crushed, to each pot. A net is used to separate the above-ground and underground parts. (3) Add flowers—Similarly, add 5.0 g of dried and crushed flowers, also dried at 60 °C, and apply a net to keep the above-ground and underground parts separated. (4) Teaching—No litter is added. Instead, simulate natural leaching by spraying the plants and allowing water to enter the pot when watering the soil. Additionally, a screen should be placed over the pots to prevent any litter from contaminating the potting soil. (5) Natural growth—No litter or screens are added, allowing the plants to grow naturally.

After 15 days of treatment, we collected rhizosphere soil (2–4 cm depth) and tested the concentration of Flavonoids.

### 4.8. KEGG Function Analysis

The PICRUSt2 software package v.2.2.0, which stored the COG and KO information corresponding to the green gene ID, was used to predict the functions of the KEGG orthology pathways. This involved knowing the metabolic pathways of the microorganisms in the sample and obtaining an abundance table of functional genes for each sample.

The analysis aimed to consider the influence of the number of copies of the 16S marker gene in the species’ genome; we also obtained the COG family and KEGG Orthology (KO) information for each OTU and calculated the abundance of each COG KO. According to the COG database, each COG and its function information could be parsed from the eggNOG database to obtain the functional abundance spectrum. Based on the KEGG database, information on KO, pathway, and EC could be obtained, and the abundance of each functional category could be determined with the OTU Abundance calculation. In addition, for the pathway, PICRUST was used to receive 3 levels of metabolic pathway information and construct an abundance table of each level.

### 4.9. Data Analysis

Flavonoid compounds were analyzed under monomer MRM conditions using a gradient elution program, with data processed through the Targetlynx v4.1 software. Soil microbial community data (16S rRNA and ITS) were analyzed via the Majorbio platform (https://cloud.majorbio.com/page/project/overview.html, accessed on 4 March 2021), and IBM SPSS Statistics 21.0 was used for significance testing. Data were further analyzed for significance and correlation using Origin software 2021, while figures were created with Excel 2019 and Prism online software (https://www.ncbi.nlm.nih.gov/tools/primer-blast/, 27 March 2019). Metabolic pathway predictions were conducted with the PICRUSt2 software package v.2.2.0, using Greengenes ID-based COG and KO data.

## 5. Conclusions

Our study highlights the significant role played by *M. micrantha* flavonoid-rich root exudates in shaping soil microbial communities. The identified flavonoids, including Mikanin, Isorhamnetin, and Hispidulin, not only promote the growth of beneficial soil microorganisms such as AMF and Achromobacter, but also enhance nitrogen cycling and nutrient availability. These microbial shifts contribute to the plant’s invasion success by creating a more favorable soil environment. Our findings suggest that by understanding and potentially harnessing the role of flavonoids and their effects on soil microbes, we can develop a novel approach to mitigate the ecological impacts of M. micrantha. This approach is not only grounded in the science of plant–microbe interactions, but also holds promise for the development of sustainable and ecologically sensitive management strategies.

## Data Availability

The dataset is available upon request from the authors.

## Supplementary Materials

The following supporting information can be downloaded at: https://www.mdpi.com/article/10.3390/ijms26010064/s1, Genome assemblies have been deposited in the China National GeneBank Database (CNGBdb, https://db.cngb.org/search/project/CNP0006652/, accessed on 17 December 2024) with CNGB Project ID CNP0006652.

## Abbreviation

UPLC-MS—Ultra-high Performance Liquid Chromatography–Mass Spectrometry; KEGG—Kyoto Encyclopedia of Genes and Genomes; COG—Clusters of Orthologous Groups; KO—Kyoto Encyclopedia of Genes and Genomes Orthology; eggNOG—Evolutionary Genealogy of Genes: Non-supervised Orthologous Groups; AMF—Arbuscular Mycorrhizal Fungi; IAA—Indole-3-acetic Acid; ABA—Abscisic Acid; MCI—Mitsubishi Chemical Industries; HPLC—High-Performance Liquid Chromatography; MRM—Multiple Reaction Monitoring; DMSO—Dimethyl Sulfoxide; NMR—Nuclear Magnetic Resonance Spectroscopy; HR-ESI-MS—High-resolution Electrospray Ionization–Mass Spectroscopy; P_Add_F—Plants Added With Flavonoids; NP_Add_F—No Plants With Flavonoids; NP_CK—No Plants With No Additives; P_CK—Plants With No Additives.

## References

[1] Day M.R. (2021). *Mikania micrantha* (bitter vine). CABI Compendium. CABI Compend..

[2] Day M.D., Clements D.R., Gile C., Senaratne W.K., Shen S., Weston L.A., Zhang F. (2016). Biology and Impacts of Pacific Islands Invasive Species. 13. *Mikania Micrantha Kunth (Asteraceae)*. Pac. Sci..

[3] Day M.D., Kawi A., Kurika K., Dewhurst C.F., Waisale S., Saul-Maora J., Fidelis J., Bokosou J., Moxon J., Orapa W. (2012). *Mikania micrantha Kunth (Asteraceae)* (Mile-a-Minute): Its Distribution and Physical and Socioeconomic Impacts in Papua New Guinea. Pac. Sci..

[4] Liu B., Yan J., Li W., Yin L., Li P., Yu H., Xing L., Cai M., Wang H., Zhao M. (2020). *Mikania micrantha* Genome Provides Insights into the Molecular Mechanism of Rapid Growth. Nat. Commun..

[5] Cai M., Chen L., Ke W., Chen M., Zhang J., Huang J., Pan Y., Peng C. (2023). Understanding the Invasion Mechanism of Malignant Alien Weed *Mikania micrantha* from the Perspective of Photosynthetic Capacity of Stems. Biol. Invasions.

[6] Xiao H. (2018). Study on Monitoring and Control of *Mikania mikania* in Nanshan Forest park, guangdong province. Rural Sci. Technol..

[7] Guo Q., Qiang S., Lin J., Yu Y. (2005). The Biological Characteristics and Integrated Management of *Mikania Micrantha*. Wuyi Sci. J..

[8] Ríos E., León A., Chávez M.I., Torres Y., Ramírez-Apan M.T., Toscano R.A., Bravo-Monzón E., Espinosa-García F.J., Delgado G. (2014). Sesquiterpene Lactones from *Mikania micrantha* and Mikania Cordifolia and Their Cytotoxic and Anti-Inflammatory Evaluation. Fitoterapia.

[9] Li P., Ling R., Peng W., Yan J. Studies on the Major Allelochemicals of the Invasive Plant *Mikania micrantha*. Proceedings of the The 5th National Congress on Invasive Biology—Invasive Organisms and Ecological Security.

[10] Xu Q., Xie H., Xiao H., Wei X. (2013). Phenolic Constituents from the roots of *Mikania micrantha* and Their Allelopathic Effects. J. Agric. Food Chem..

[11] Liu X., Zhou Y., Qi C., Li Y., Wang Q., Guo M., Yan D., Cao A. (2012). Effects of *Mikania micrantha* Invasion on soil Nutrient Contents and Enzyme Activities. Ecol. Environ. Sci..

[12] Yang Q., Liang Y., Yang J., Hu Z. (2015). Soil Microbial Characteristics in the Rhizosphere of Exotic Invasive *Mikania Micrantha*. Ecol. Sci..

[13] Chukwuebuka E., Chinenye I.J. (2015). Biological Functions and Anti-nutritional Effects of Phytochemicals in Living System. J. Pharm. Biol. Sci..

[14] Dias M.C., Pinto D.C.G.A., Silva A.M.S. (2021). Plant Flavonoids: Chemical Characteristics and Biological Activity. Molecules.

[15] Laoué J., Fernandez C., Ormeño E. (2022). Plant Flavonoids in Mediterranean Species: A Focus on Flavonols as Protective Metabolites under Climate Stress. Plants.

[16] Cesco S., Mimmo T., Tonon G., Tomasi N., Pinton R., Terzano R., Neumann G., Weisskopf L., Renella G., Landi L. (2012). Plant-Borne Flavonoids Released into the Rhizosphere: Impact on Soil Bio-Activities Related to Plant Nutrition. A review. Biol. Fertil. Soils.

[17] Wu J., Lv S., Zhao L., Gao T., Yu C., Hu J., Ma F. (2023). Advances in the Study of the Function and Mechanism of the Action of Flavonoids in plants under Environmental Stresses. Planta.

[18] Sugiyama A., Yazaki K. (2014). Flavonoids in Plant Rhizospheres: Secretion, Fate and Their Effects on Biological Communication. Plant Biotechnol..

[19] Huang X.-F., Chaparro J.M., Reardon K.F., Zhang R., Shen Q., Vivanco J.M. (2014). Rhizosphere Interactions: Root Exudates, Microbes, and Microbial Communities. Botany.

[20] Al-Kaisi M.M., Lal R., Olson K.R., Lowery B. (2017). Fundamentals and Functions of Soil Environment. Soil Health and Intensification of Agroecosytems.

[21] Wang X., Cheng L., Xiong C., Whalley W.R., Miller A.J., Rengel Z., Zhang F., Shen J. (2024). Understanding Plant–Soil Interactions Underpins Enhanced Sustainability of Crop Production. Trends Plant Sci..

[22] Stanek M., Zubek S., Stefanowicz A.M. (2021). Differences in Phenolics Produced by Invasive Quercus rubra and native Plant Communities Induced Changes in Soil Microbial Properties and Enzymatic Activity. For. Ecol. Manag..

[23] Xiong H.-H., Lin S.-Y., Chen L.-L., Ouyang K.-H., Wang W.-J. (2023). The Interaction between Flavonoids and Intestinal Microbes: A Review. Foods.

[24] Chen B.M., Peng S.L., Ni G.Y. (2009). Differential belowground allelopathic effects of leaf and root of Mikania micrantha. Biol. Invasions.

[25] Valesi A.G., Rodriguez E., Velde G.V., Mabry T. (1972). Methylated Flavonols in *Larrea Cuneifolia*. Pergamon.

[26] Yu H., Le Roux J.J., Jiang Z., Sun F., Peng C., Li W. (2021). Soil Nitrogen Dynamics and Competition during Plant Invasion: Insights from *Mikania micrantha* Invasions in China. New Phytol..

[27] Poudel M., Adhikari P., Thapa K. (2019). Biology and Control Methods of The Alien Invasive Weed Mikania. Environ. Contam..

[28] Zomborszki Z.P., Kúsz N., Csupor D., Peschel W. (2019). Rhodiosin and Herbacetin in Rhodiola rosea preparations: Additional Markers for Quality Control?. Pharm. Biol..

[29] Magar R.T., Sohng J.K. (2020). A Review on Structure, Modifications and Structure-Activity Relation of Quercetin and Its Derivatives. J. Microbiol. Biotechnol..

[30] Wang G., Cao F., Chang L., Guo X., Wang J. (2014). Temperature Has More Effects than Soil Moisture on Biosynthesis of Flavonoids in *Ginkgo (Ginkgo biloba* L.) Leaves. New For..

[31] Li Y., Chen S., Li C., Deng S., Gu W. (2008). Isolation and Identification of Allelochemicals from *Mikania micrantha*. J. S. China Agric. Univ..

[32] Cooper J.E. (2004). Multiple Responses of Rhizobia to Flavonoids During Legume Root Infection. Adv. Bot. Res..

[33] Hassan S., Mathesius U. (2012). The Role of Flavonoids in Root-Rhizosphere Signalling: Opportunities and Challenges for Improving Plant-Microbe Interactions. J. Exp. Bot..

[34] Sugiyama A. (2021). Flavonoids and Saponins in Plant Rhizospheres: Roles, Dynamics, and the Potential for Agriculture. Biosci. Biotechnol. Biochem..

[35] Weston L.A., Mathesius U. (2013). Flavonoids: Their Structure, Biosynthesis and Role in the Rhizosphere, Including Allelopathy. J. Chem. Ecol..

[36] Wang L., Chen M., Lam P.Y., Dini-Andreote F., Dai L., We Z. (2022). Multifaceted roles of flavonoids mediating plant-microbe interactions. Microbiome.

[37] Tian B., Pei Y., Huang W., Ding J., Siemann E. (2021). Increasing Flavonoid Concentrations in Root Exudates Enhance Associations between Arbuscular Mycorrhizal Fungi and an Invasive Plant. ISME J..

[38] Chen L., Li H., Yang M., Wan F. Using PCR-DGGE Technology to Analyze Bacterial Communities in the Rhizosphere Soil of Invasive Plants Mikania micrantha and Bidens clover. Proceedings of the Third National Biological Invasion Conference.

[39] Mager D., Thomas A. (2011). Extracellular Polysaccharides from *cyanobacterial* Soil Crusts: A Review of Their Role in Dryland Soil Processes. J. Arid. Environ..

[40] Li Z., Xiao J., Chen C., Zhao L., Wu Z., Liu L., Cai D.P. (2020). Promoting Desert Biocrust Formation Using Aquatic *cyanobacteria* with the Aid of MOF-based Nanocomposite. Sci. Total Environ..

[41] Hannula S., Morriën E., van der Putten W., de Boer W. (2020). *Rhizosphere* Fungi Actively Assimilating Plant-Derived Carbon in a Grassland Soil. Fungal Ecol..

[42] Gao P., Zhang X., Huang X., Chen Z., Marietou A., Holmkvist L., Qu L., Finster K., Gong X. (2023). Genomic Insight of Sulfate Reducing Bacterial Genus *Desulfofaba* Reveals Their Metabolic Versatility in Biogeochemical Cycling. BMC Genom..

