# Supplementary material for: Influence of Mikania micrantha Kunth Flavonoids on Composition of Soil Microbial Community
Source: Int J Mol Sci. 2024 Dec 25;26(1):64. doi: 10.3390/ijms26010064 (PMC11720020; doi:10.3390/ijms26010064)
Supplement: Supplementary file 1 [file ijms-26-00064-s001.zip › ijms-3357865-supplementary.pdf]

Influence of *Mikania micrantha* Kunth Flavonoids on Composition of Soil Microbial Community

Qilin Yang[1], Wenyang Cui[1], Zijun Guan[1], Zhenzhen Wang[1], Israt Jahan[1], Ping Li[1, *], Feng Qin[2], Xi Qiao[2], Bo Liu[2], and Jian Yan[1, *]

Supporting Information

S1

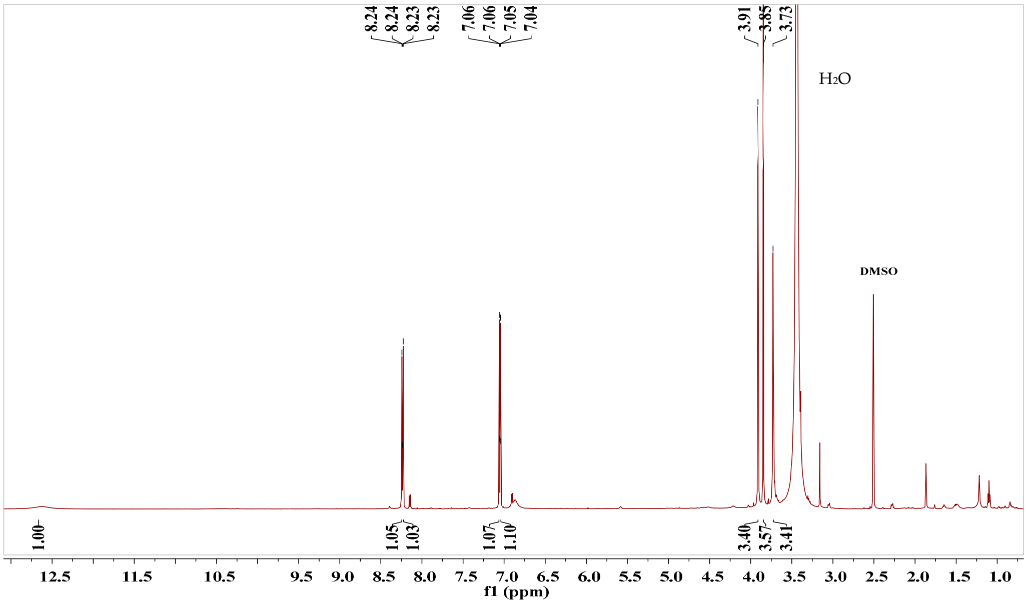

Figure S1. Mikanin 1H-NMR

S2

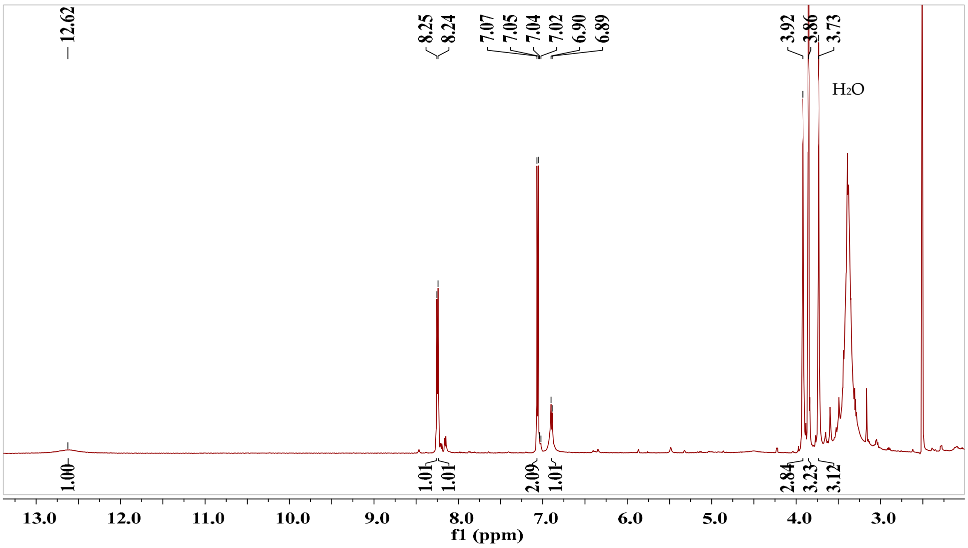

Figure S2. Tambulin 1H-NMR

S3

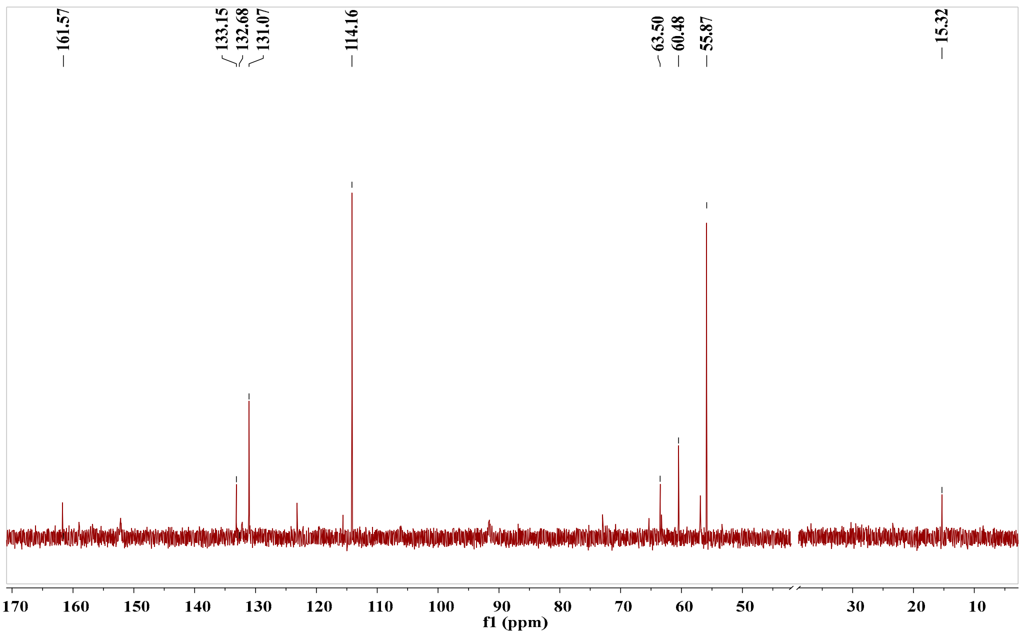

Figure S3. Tambulin 13C-NMR

S4

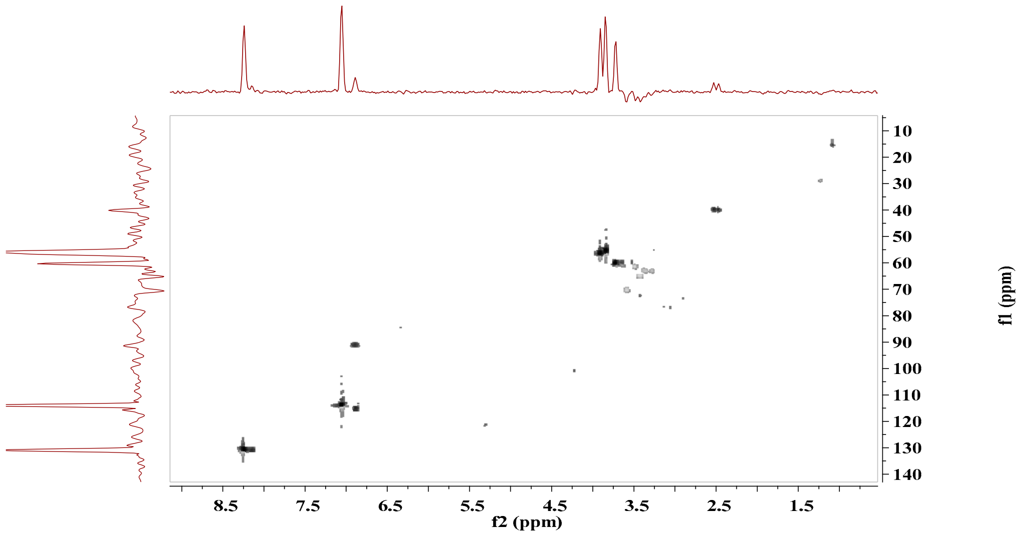

Figure S4. Tambulin HMBC

S5

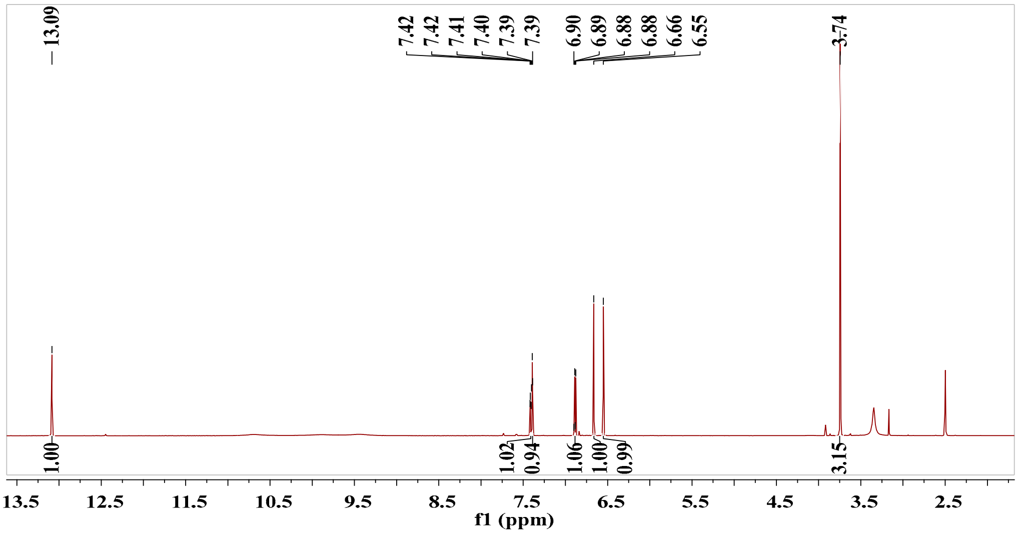

Figure S5. Isohamnetin 1H-NMR

S6

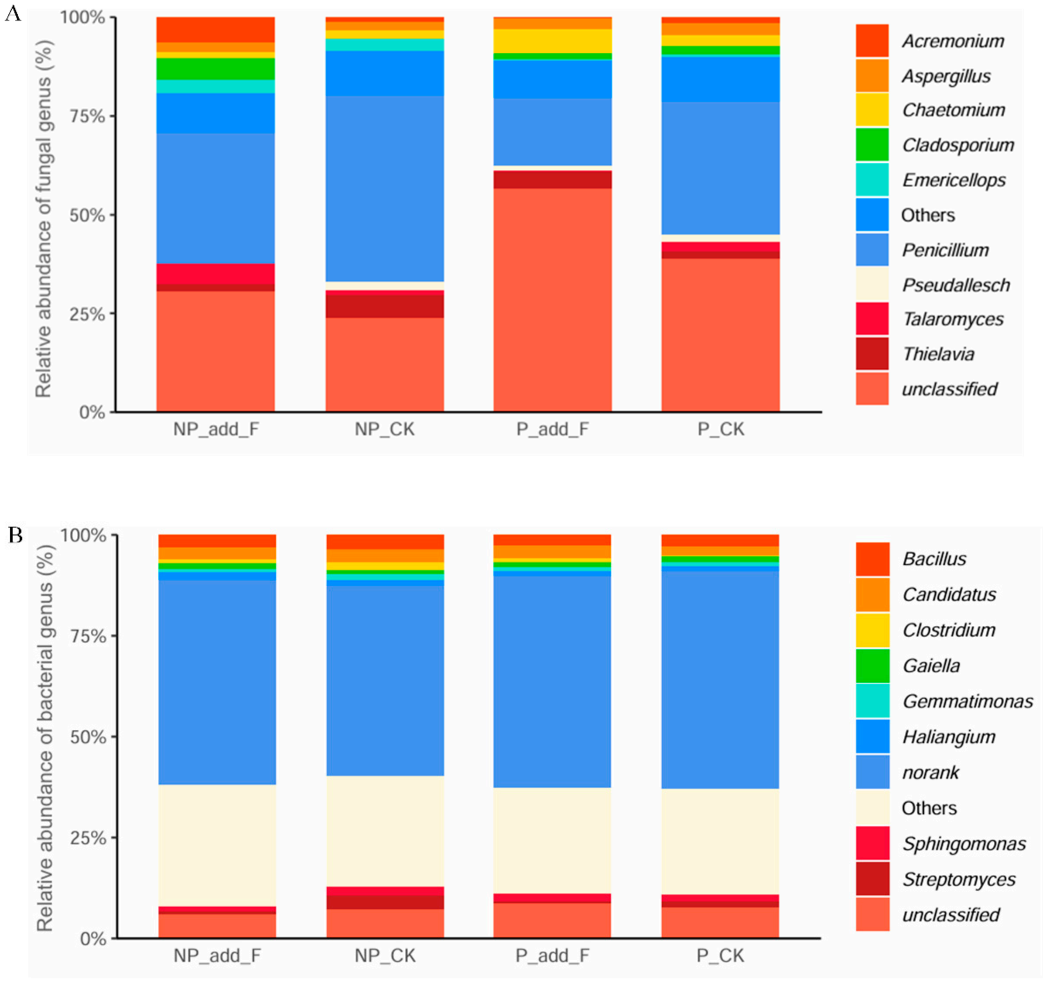

Figure S6. Relative abundance of the dominant fungal (A) and bacterial (B) taxa in different treatments.
